# Television habits in relation to overweight, diet and taste preferences in European children: the IDEFICS study

**DOI:** 10.1007/s10654-012-9718-2

**Published:** 2012-08-22

**Authors:** Lauren Lissner, Anne Lanfer, Wencke Gwozdz, Steingerdur Olafsdottir, Gabriele Eiben, Luis A. Moreno, Alba M. Santaliestra-Pasías, Éva Kovács, Gianvincenzo Barba, Helle-Mai Loit, Yiannis Kourides, Valeria Pala, Hermann Pohlabeln, Stefaan De Henauw, Kirsten Buchecker, Wolfgang Ahrens, Lucia Reisch

**Affiliations:** 1 Public Health Epidemiology Unit, Department of Community Medicine and Public Health, Sahlgrenska Academy, University of Gothenburg, Box 454, 405 30 Gothenburg, Sweden; 2 BIPS - Institute for Epidemiology and Prevention Research, Bremen, Germany; 3 Copenhagen Business School, Copenhagen, Denmark; 4 Department of Food and Nutrition, and Sport Science, University of Gothenburg, Gothenburg, Sweden; 5 GENUD (Growth, Exercise, Nutrition and Development) Research Group, School of Health Sciences, University of Zaragoza, Zaragoza, Spain; 6 University of Pécs, Pécs, Hungary; 7 Epidemiology & Population Genetics, Institute of Food Sciences, CNR, Avellino, Italy; 8 National Institute for Health Development, Tallinn, Estonia; 9 Research and Education Institute of Child Health, Strovolos, Cyprus; 10 Epidemiology and Prevention Unit, Department of Preventive & Predictive Medicine, Fondazione IRCCS Istituto Nazionale dei Tumori, Milan, Italy; 11 Department of Public Health, Ghent University, Ghent, Belgium; 12 Department of Food Science, TTZ, Bremerhaven, Germany

**Keywords:** Television, Diet, Taste preference, Childhood overweight

## Abstract

Early television exposure has been associated with various health outcomes including childhood obesity. This paper describes associations between patterns of television viewing, on one hand, and diet, taste preference and weight status, on the other, in European preschoolers and schoolchildren. The IDEFICS baseline survey was conducted at examination centers in Italy, Estonia, Cyprus, Belgium, Sweden, Germany, Hungary, and Spain. 15,144 children aged 2–9 completed the basic protocol, including anthropometry and parental questionnaires on their diets and television habits. A subsample of 1,696 schoolchildren underwent further sensory testing for fat and sweet taste preferences. Three dichotomous indicators described: children’s habitual television exposure time; television viewing during meals; and having televisions in their bedrooms. Based on these variables we investigated television habits in relation to overweight (IOTF) and usual consumption of foods high in fat and sugar. A possible role of taste preference in the latter association was tested in the sensory subgroup. All television indicators were significantly associated with increased risk of overweight, with odds ratios ranging from 1.21 to 1.30, in fully adjusted models. Children’s propensities to consume high-fat and high-sugar foods were positively and, in most analyses, monotonically associated with high-risk television behaviors. The associations between television and diet propensities were not explained by preference for added fat or sugar in test foods. To summarize, in addition to being more overweight, children with high-risk television behaviors may, independent of objectively measured taste preferences for fat and sugar, passively overconsume higher-fat and particularly higher-sugar diets.

## Introduction

Early exposure to television (TV) is associated with a number of physical and psychological health outcomes, including weight problems. The American Academy of Pediatrics recommended in 2001 that viewing time be limited to 1–2 h per day of quality programming and that televisions be removed from children’s bedrooms [1]. More restrictive limits are sometimes applied to pre-school children; for instance in Australia it is recommended that 2–5 year olds should be limited to less than 1 h per day of sitting and watching television and the use of other electronic media [2].

Obesity is one of the more widely discussed examples of television’s negative health consequences. There is extensive literature on associations between television viewing habits and childhood obesity, including observational studies with cross-sectional and prospective designs [3, 4], as well as interventions [5, 6]. It is not well understood whether the relation between TV viewing and obesity depends primarily on increased food intake or decreased energy expenditure [6, 7]. Specific mechanisms for effects of television on obesity include exchange of vigorous activity patterns with sedentary ones during television viewing time and passive overconsumption of food due to disruption of dietary intake regulation by the television. Additionally, direct triggering effects of televised food promotions on eating behaviors have been investigated and it has been proposed that food and taste preferences may in part be determined by messages seen on television [8, 9]. An updated review of the extent and effects of food promotion on children was prepared for WHO [10], concluding that the evidence for links to food behaviors and diet-related health outcomes are modest but likely to be causal. Beyond direct influences of television, it is likely that diet patterns differ in children with different TV viewing habits due to underlying social factors affecting the lifestyle and environment. Several aspects of this problem have received relatively less attention, i.e. cross-cultural aspects of television viewing habits in relation to overweight and obesity, and whether associations are explained by underlying differences in diet, taste preferences, socioeconomic status and other aspects of lifestyle.

This cross-sectional study describes associations between patterns of television viewing, on one hand, and overweight, diet, and taste preference, on the other, in children from eight European regions with diverse television and food cultures and varying prevalence of obesity and overweight. It is hypothesized that children’s television habits are associated with both weight status and dietary quality, independent of regional and socioeconomic variations. The secondary hypothesis is that the association between television habits and habitual consumption of foods high in sugar and fat can be explained by children’s taste preferences for sugar and fat. In all analyses we will compare: children who usually watch television during meals versus those who do not; children who regularly watch at least 1 h of TV per day and those who watch less; and children with and without television or video/DVD in their bedrooms. Based on these three factors, we will first examine whether children’s television habits as described above are related to weight status including whether the association is consistent in the 8 survey countries and whether the association can be explained by other differences within and between survey countries. In the subsequent sections of this paper we examine whether children’s television habits are related to indicators of high-fat and high-sugar dietary patterns and finally whether the association can be explained by children’s individual taste preferences for fat and sugar, respectively.

## Methods

### Study design

This report is based on data from the IDEFICS study (“IDentification and prevention of dietary and lifestyle induced health EFfects In Children and infantS”). The IDEFICS study, supported by the sixth framework program of the European Commission, is a multi-center cohort and intervention study with a focus on diet-related health outcomes. Data collection methods were standardized across all study centers, as previously documented [11]. The IDEFICS baseline survey was conducted between September 2007 and June 2008 in examination centers established by investigators in Italy, Estonia, Cyprus, Belgium, Sweden, Germany, Hungary and Spain. All survey centers obtained approval from their local research ethics committees or institutional review boards.

Participants were between 2 and 9 years old and recruited via their daycare centers or schools. The examination protocol included a detailed questionnaire in which parents described their children’s lifestyles, diets, and family circumstances. At the same time a physical examination of all children was conducted to characterize weight status and other indicators of cardiometabolic health. Informed consent was obtained from children’s parents, one of whom was usually present at the examination. During the baseline survey, 16,864 children were examined (53.5 % of those invited), of whom 16,220 met basic inclusion criteria of complete information on weight, height, age and sex. After 2 years, the examination was repeated. Between these examinations, half of the participants underwent community oriented interventions designed to promote healthy lifestyles in children and families [13]. The present analysis is limited to baseline data collected prior to the intervention, with a total analytical sample of 15,144, corresponding to the number of children who met inclusion criteria and also had data on at least one of the television viewing variables used in this analysis.

### Weight status

Children’s body weight was measured to the nearest 0.1 kg in the morning in fasting condition and wearing underwear (TANITA BC 420 SMA scale). Height was measured to the nearest 0.1 cm (SECA 225 stadiometer). Based on these measures, BMI was calculated and for the purpose of the present analysis, children were classified as overweight (including obese) or non-overweight (including underweight) using age and sex-specific cut-points for overweight defined by Cole [12].

### Television exposure

Television viewing habits were described in the parental questionnaires, which were completed by a parent or guardian living with the child. The first question used here was “How long does your child usually watch TV/video/DVD per day?” with separate answers given for weekdays and Saturday/Sundays. For this variable, responses were dichotomized according to whether the response was less than 60 min per day on weekdays or weekend days, or at least 60 min on weekdays and/or weekend days. Alternative responses of 30 and 120 min as indicators of high-risk television exposure were also tested. The second question was “Does your child watch TV at meals?” with possible responses of never, rarely, sometimes, often and always. Those children whose parents gave responses of never or rarely were classified as not watching TV during meals, while others were classified positive in this respect. The third variable is based on whether or not the child had a TV and/or DVD or video player in the bedroom. These classifications are abbreviated in this paper as TV60, EATTV, and TVROOM and referred to collectively as “high risk” TV behaviors. This is based in part on previously mentioned recommendations [1, 2] to limit children’s television to 1–2 h per day and remove TVs from bedrooms. The question about watching television during mealtimes is included as an additional risk factor here, due to our special focus on diet preferences, and in view of the literature on effects of televised messages on children’s eating behaviors [8, 9, 10]. All three questions were included in a pilot study testing reproducibility of the parental questionnaire, and the results suggest agreement between repeat measures of the three TV variables used here (Cohen’s kappas: k = 0.85 (95 % CI: 0.78–0.91), k = 0.56 (95 % CI: 0.47–0.65), k = 0.67 (95 % CI: 0.57–0.76), for TVROOM, TV60 and EATTV respectively).

### Dietary habits

Usual consumption of high-fat and sweet foods and beverages was assessed based on parental responses to the Children’s Eating Habits Questionnaire (CEHQ). This questionnaire was developed as a screening tool to investigate usual food consumption and behaviors associated with weight status and general health in children. The food frequency questionnaire (FFQ) within the CEHQ describes frequency of the child’s consumption of 43 food items on a typical week during the preceding 4 weeks, excluding foods provided in school or daycare settings. To maintain comparability across countries, the same foods and beverages were translated into 8 languages, but for some items country-specific examples were also noted. The CEHQ-FFQ was designed to reflect intake of foods that are believed to be obesity-promoting or inhibiting, but does not aim to assess total food or energy intake. A complete list of foods in the CEHQ-FFQ and their reproducibility has been reported previously [14]. Correlations between calcium and potassium excretion in spot urine samples in relation to estimated milk consumption were highly significant and indicated acceptable validity of the CEHQ-FFQ for milk products [15]. In the present analysis, children for whom more than 21 (50 %) of the FFQ answers were missing or not known were excluded. When creating composite scores, missing food items were treated as not consumed. We calculated the weekly consumption frequencies of each of 17 foods and beverages that are high in fat and 12 foods and beverages with high sugar content, based on which a weekly frequency was calculated for each of these categories. The remaining items in the 43-item questionnaire were also converted into weekly frequency scores. A continuous index was developed, using the total weekly frequency for the high-sugar or high-fat items divided by the individual’s total consumed food frequencies. These latter indices, also referred to here as fat or sugar propensity ratios, as described previously [16] were considered to reflect proportions in the whole diet. The rationale for using a ratio in this analysis is to provide a description of a child’s diet quality, standardizing on number of food items reported and frequency of consumption. In this way we avoid misclassifying children as high-sugar or high-fat consumers simply because they consume all types of food frequently, while also correcting for variation in the number of meals and eating occasions away from home and thus not captured in the FFQ. As an example, a propensity score of 0.33 indicates that one-third of weekly food items consumed were in the high-fat (or high-sugar) group; this number was approximately the top quartile cut-point for both food propensity scores. Relative validity of this approach was explored by relating usual propensity for high-sugar and high-fat foods, respectively, to energy-adjusted sugar and fat intakes calculated from one 24-h recall interview, which was also assessed in the majority of the children by parental proxy. Highly significant monotonic trends (F = 144.1, *p* < 0.0001 for sugar and F = 21.2, *p* < 0.0001 for fat) indicated agreement between these different approaches to ranking children on their sugar and fat intakes (unpublished results).

### Parental education and other covariates

As possible explanatory factors for associations between television, diet, and obesity, we included parental education as a covariate. The highest educational level attained by either parent was used as an indicator of socioeconomic status and considered in all models as a covariate. To improve cross-country comparability of the education variable, it was coded according to the International Standard Classification of Education (ISCED) [17]. The original ISCED levels 1, 2, and 3 were categorized as lower educational attainment and level 4 and above as higher educational attainment. Sensitivity analyses were conducted, adjusting for a standardized (9-level) income variable rather than education as a socioeconomic indicator. These results are not shown because use of this variable did not affect any of the results or conclusions. Additionally, in analysis with weight status as dependent variable, we further adjusted for two indicators of physical activity. All parents were asked to report their children’s usual patterns of outdoor play and indoor sports, based on which a physical activity indicator was created. 6,799 of these children also wore accelerometric devices, and a variable was calculated reflecting moderate and vigorous physical activity time [18]. In these children, the same analyses of weight status in relation to television habits were repeated after adjusting for measured physical activity levels instead of parental reports.

### Taste preference tests

A subsample of 6–9 year old children later underwent sensory testing in the schools, as reported previously [16]. 1,696 schoolchildren participated in at least one supplementary test for sweet or fat preference. The children chose between a high-versus low-fat cracker and a natural- versus sugar-sweetened juice. Each pair of food samples was presented to each child on a game board. Children tasted both samples and then placed their preferred sample on a “smiley” on top of the board. Afterwards they rinsed their mouths with water and proceeded with the following pair of samples. Fat preference tests were carried out in all 8 survey centers, while sweet preference tests were not done in Cyprus because children proved to be unfamiliar with apple juice. These data were used to study whether taste preference may be driving associations between food choice and television habits.

### Statistics

In the first analysis, logistic models were used to calculate prevalence odds ratios for overweight (including obesity) as a function of the three television indicators, with one model adjusting for survey country (dummy variables), parental education (dichotomous), age (continuous) and sex. In a fully adjusted model, the two diet indicators (fat and sugar propensity ratios) were simultaneously added as continuous covariates to the previous model, and additionally mutual adjustment was done for the other 2 television variables and physical activity. A figure was used to illustrate individual country-specific odds ratios adjusted for age, sex and education, together with a pooled result that further adjusted for country. In the second analysis, logistic regression was used to describe odds of each television habit in relation to fat or sweet propensity (both ratios converted to quartiles), after adjustment for age, sex, socioeconomic status and survey country studied. Due to known between-country differences in key variables included, mixed models as well as fixed effects models were tested. Both methods yielded similar results and in this report survey countries are treated as fixed effects. Interactions were tested to determine if associations differed in preschool versus school-aged children. Prevalence odds ratios (OR) are reported with 95 % confidence intervals. Sensitivity analyses were done testing different cut-points for the variable reflecting usual television exposure time. In the subsample with sensory data, we compared the relation between diet propensities and TV habits before and after adjusting for preference for fat or sweet in test foods, respectively. Analyses and figures were done with the Statistical Analysis System, SAS, version 9.2.

## Results

Table 1 presents basic data on age, education, gender and setting in the full sample and the sub-sample that underwent sensory testing. Country-based descriptive statistics on television, weight status, and diet are then shown for the full sample of boys and girls separately in Table 2. The two countries with least overweight also had the lowest rates of eating meals while watching TV, while the pattern was less apparent for viewing time and for television in bedroom. The survey center in Italy, with the highest rates of overweight and obesity, also had the highest proportion of children who watch TV during meals and have TVs in their bedrooms, although their usual viewing time was close to the 8-country average. Regarding diet, the most notable difference across survey centers was a low propensity for consumption of high-sugar foods and beverages by Swedish children. Based on the uncertainties inherent in this type of ecological observation, all formal analyses were based on individual-level data. To take into consideration the large country-level differences across survey centers, pooled analyses of associations between individual television habits, on one hand, and weight status and diet preferences on the other, are adjusted for country in which the survey was conducted as described previously.

**Table 1:** Selected sociodemographic characteristics of full sample and sub-sample participating in taste preference tests

Survey center	Full sample					Sub-sample			
	N^a^	Age (SD)	Education %^b^	% Girls	Setting^c^	N^d^	Age (SD)^e^	Education^b^	% Girls
Italy	2,247	6.1 (1.8)	18.3	48.2	56.7	271	7.6 (0.8)	17.4	46.1
Estonia	1,666	5.9 (2.1)	57.5	50.8	49.2	282	7.9 (0.4)	60.4	56.4
Cyprus^f^	1,646	6.1 (1.3)	82.4	49.1	59.5	81	7.0 (0.7)	92.5	55.6
Belgium	1,860	5.7 (1.6)	68.3	49.0	45.8	159	7.2 (0.7)	72.4	49.7
Sweden	1,759	5.7 (2.0)	79.6	48.5	49.2	156	7.6 (0.8)	78.1	50.0
Germany	2,000	6.2 (1.8)	37.1	49.1	57.4	228	7.6 (0.8)	37.7	57.0
Hungary	2,499	6.3 (1.8)	52.9	49.9	59.0	240	7.9 (0.6)	55.0	50.4
Spain	1,467	5.8 (1.8)	62.9	48.5	52.8	195	7.1 (0.6)	67.5	45.1
All	15,144	6.0 (1.8)	55.2	49.1	54.1	1,612	7.6 (0.7)	54.5	51.2

^a^N for participants with at least one TV variable, totals vary slightly by analysis

^b^% with ISCED 4–6 (highest level attained by either parent)

^c^Percent schoolchildren (compared to daycare)

^d^N for participants with at least one TV variable, full information on diet and at least one taste preference test completed

^e^All children in school setting

^f^Not included in sugar preference test

**Table 2:** Key study variables by sex, in 15,144 boys and girls aged 2–9 years included in full sample

Survey center	N	Boys						Girls						
		% OWOB^a^	% EATTV^b^	% TV60^c^	% TV ROOM^d^	Fat ratio (SD)^e^	Sugar ratio (SD)^f^	N	% OWOB^a^	% EATTV^b^	% TV60^c^	% TV ROOM^d^	Fat ratio (SD)^e^	Sugar ratio (SD)^f^
Italy	1,164	39.9	80.2	48.9	69.7	0.24 (0.11)	0.29 (0.13)	1,083	44.3	73.3	45.5	64.2	0.23 (0.10)	0.28 (0.13)
Estonia	819	13.6	56.4	58.4	33.1	0.27 (0.08)	0.25 (0.10)	847	14.9	55.5	55.1	34.2	0.27 (0.08)	0.25 (0.09)
Cyprus	838	20.4	74.1	58.7	31.2	0.25 (0.10)	0.23 (0.10)	808	23.6	67.1	54.4	26.0	0.24 (0.09)	0.22 (0.10)
Belgium	948	7.7	29.2	48.0	11.1	0.29 (0.10)	0.33 (0.11)	912	9.5	27.5	38.0	9.7	0.29 (0.09)	0.31 (0.11)
Sweden	906	9.5	27.8	48.6	24.7	0.22 (0.09)	0.14 (0.08)	853	11.8	28.0	44.7	19.9	0.22 (0.09)	0.13 (0.07)
Germany	1,019	13.4	32.5	40.5	24.5	0.29 (0.09)	0.30 (0.12)	981	18.5	29.5	39.2	21.6	0.29 (0.09)	0.29 (0.11)
Hungary	1,253	16.0	41.0	42.1	46.0	0.26 (0.09)	0.26 (0.12)	1,246	18.0	38.0	37.9	45.1	0.27 (0.08)	0.26 (0.11)
Spain	755	17.7	57.2	34.1	9.5	0.24 (0.08)	0.26 (0.09)	712	23.6	56.5	29.5	8.3	0.23 (0.09)	0.25 (0.09)
All	7,702	17.9	48.7	47.1	33.5	0.26 (0.10)	0.26 (0.12)	7,442	20.9	45.7	42.8	30.9	0.26 (0.09)	0.25 (0.12)

N for participants with at least one TV variable, totals vary slightly by analysis

^a^OWOB: percent overweight or obese, IOTF

^b^EATTV: percent who regularly eat meals while watching TV

^c^TV60: Percent who watch TV at least 60 min per day weekends or weekdays

^d^TVROOM: Percent with TV/DVD/video in bedroom

^e^Propensity to consume items high in fat, relative to frequency of all items on FFQ

^f^Propensity to consume items high in sugar, relative to frequency of all items on FFQ

The first analysis series examined whether television viewing patterns were associated with weight status. The television indicators were significantly related to increased odds for overweight (Table 3), with only minor variation in magnitude of the association in girls and boys. Eating while watching television was significantly associated with overweight, with prevalence odds ratios of 1.20 (95 % CI 1.04–1.40) for boys and 1.35 (95 %CI 1.17–1.55) for girls in fully adjusted models. Having a television in the bedroom showed similar associations: OR 1.39 (1.19–1.61) in boys and 1.23 (1.06–1.42) in girls. Finally, watching 60 min a day or more on weekdays and/or weekends was associated with overweight to a similar degree in both sexes: OR 1.20 (1.05–1.38) in boys and OR 1.21 (1.06–1.38) in girls. These estimates were all derived from statistical models that adjusted for age, survey country, parental education, dietary propensities, parental reports of physical activity, and the other two television variables. Although not the focus of this paper, major differences across survey countries were observed for prevalence of overweight, and high parental education was a protective factor. Both of these latter results were statistically independent of the television viewing parameters under investigation here. No effect modification by age group was observed. Sensitivity analyses were also conducted using different cut-points for television viewing, i.e. rather than 60 min per day, we tested 30 min per day and 2 h per day on weekdays and/or weekends. Irrespective of the definition used, children in the higher exposure category had greater odds of overweight and there was no statistical advantage to either raising or lowering the cut-point. All of the high-risk TV indicators were significantly associated (positively) with child’s age in years and (negatively) with higher parental education; data not shown. Finally we repeated the analysis in the subset of children with accelerometry data and observed that if the positive association between television and obesity reported in Table 3 was hardly changed after adjusting for objectively measured physical activity levels as opposed to proxy reported activity.

**Table 3:** Prevalence odds ratios (OR) for overweight associated with (a) eating while watching TV never or rarely vesus more frequently (b) watching TV on average at least 60 min per day on weekdays and/or weekends; and (c)having a TV/video/DVD player in bedroom

TV exposure variable	Odds ratio (OR) for overweight		Odds ratio (OR) for Overweight	
	Reduced model^a^		Fully adjusted model^b^	
	OR	95 % CI	OR	95 % CI
(A) EATTV: regularly eat meals while watching TV				
Boys	1.18	1.03–1.36	1.20	1.04–1.40
Girls	1.36	1.19–1.54	1.35	1.17–1.55
Both	1.27	1.16–1.40	1.28	1.16–1.42
(B) TV60: at least 60 min/day average weekdays or weekends				
Boys	1.24	1.10–1.41	1.20	1.05–1.38
Girls	1.24	1.10–1.40	1.21	1.06–1.38
Both	1.24	1.14–1.36	1.21	1.10–1.33
(C) TVROOM: has a TV or video/DVD in bedroom				
Boys	1.44	1.25–1.66	1.39	1.19–1.61
Girls	1.24	1.08–1.42	1.23	1.06–1.42
Both	1.33	1.20–1.47	1.30	1.17–1.44

Including obesity, Cole (2000)

^a^Reduced model includes TV exposure variable with age (continuous), survey country (fixed effect), parental education (higher versus lower), and sex where not stratified

^b^Fully adjusted model includes variables in reduced model plus dietary fat propensity and sugar propensity (continuous ratios) and other two television variables and the children’s usual physical activity level

**Fig. 1:**
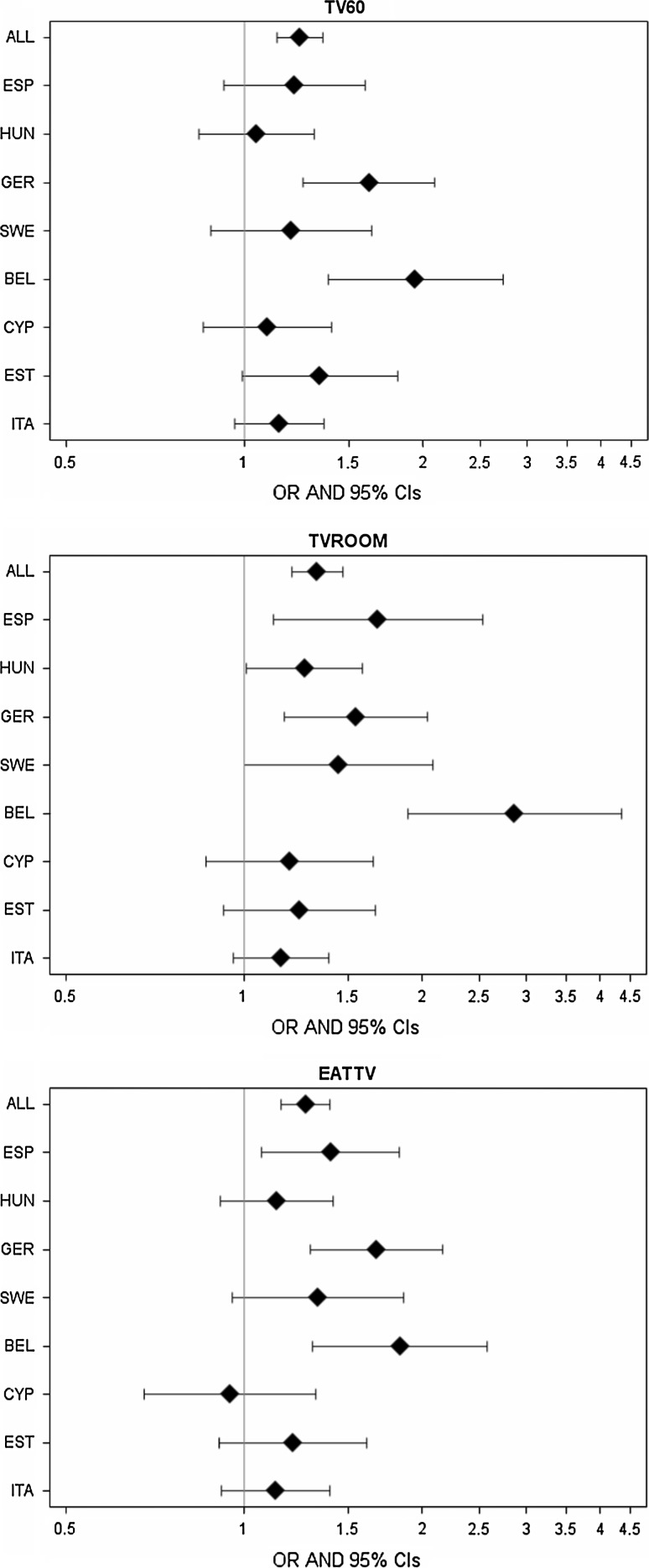
Odds ratios for overweight (including obesity) in 8 survey centers and in all countries combined. All analyses considered covariates age, sex, and parental education level; the pooled estimate (ALL) further adjusts for survey country. Each panel describes one television-related exposure (TV60: watching TV at least 60 min/day on weekdays or weekends; TVROOM: having a television/video/DVD in bedroom; and EATTV: eating while watching TV)

Country-specific analyses, illustrated in Fig. 1, indicated that the magnitude of the associations between television habits and overweight varied among different countries. While not all associations were statistically significant, all point estimates except for Cyprus indicated positive associations.

We next investigated whether a propensity to eat high-fat foods or high-sugar foods was associated with television habits. We found that television viewing patterns differed significantly among children with different dietary propensity scores, with strong indications of a linear gradient (Table 4). Eating while watching TV was associated with a higher proportion of high-fat items and high-sugar items in the diet, in proportion to total number of foods consumed. This association was independent of education, age, sex, and country. Similar associations were observed for watching TV more than 60 min per day and having a TV in the bedroom, in relation to increasing propensity for high-fat and high-sugar foods. All three television indicators tended to be more strongly increasing across quartiles for sugar propensity ratios, compared with corresponding quartiles for dietary fat. Similar associations were seen in boys and girls. No effect modification by age group was observed.

**Table 4:** Relation between fat/sugar propensity (quartiles) and television habits, prevalence odds ratios (95 % CI) adjusted for age, sex, survey center, and parental education

Odds ratio for television habit, by quartile of	Fat propensity^a^				Sugar propensity^b^			
	Q1	Q2	Q3	Q4	Q1	Q2	Q3	Q4
Television variables								
EATTV: Regularly eat meals while watching TV	1	1.10 (0.99–1.22)	1.36 (1.23–1.51)	1.49 (1.34–1.65)	1	1.29 (1.16–1.43)	1.50 (1.35–1.69)	1.93 (1.72–2.16)
TV60: At least 60 min/day average weekdays or weekends	1	1.14 (1.03–1.25)	1.22 (1.11–1.35)	1.43 (1.29–1.57)	1	1.22 (1.11–1.35)	1.40 (1.26–1.55)	1.84 (1.66–2.05)
TVROOM: Has a TV or video/DVD in bedroom	1	1.01 (0.90–1.13)	1.12 (1.00–1.25)	1.20 (1.07–1.35)	1	1.16 (1.03–1.31)	1.39 (1.23–1.56)	1.74 (1.54–1.97)

^a^Quartiles for dietary fat propensity were calculated as ratio of fried potatoes, whole fat milk, whole fat yogurt, fried fish, cold cuts/sausages, fried meat, fried eggs, mayonnaise, cheese, chocolate- or nut-based spread, butter/margarine on bread, nuts/seeds/dried fruit, salty snacks, savory pastries, chocolate-based candies, cake/pudding/cookies, and ice cream to total frequencies/week. Quartile cutpoints for propensity ratio were 0.191, 0.251, 0.318

^b^Quartiles for sugar propensity were calculated as of fruit with added sugar, fruit juice, sugar-sweetened drinks, sweetened breakfast cereals, sweetened milk, sweetened yogurt, jam/honey, chocolate- or nut-based spread, chocolate-based candies, non-fat candies, cake/pudding/cookies, and ice cream ratio to total frequencies/week. Quartile cutpoints for propensity ratio were 0.170, 0.247, 0.330

Lastly, we explored the association between the diet propensity ratios and TV habits in the sub-sample that underwent sensory testing. For simplicity we used the 4-level indicator here based on the quartile cut-points developed for the previous analysis. The main results in this subsample were similar to those seen in the full sample, although fat intake was only significantly associated with one of the TV variables (eating while watching TV). In contrast, the results for sugar propensity were robust using all three television indicators. Controlling for a preference for the sweet taste did not materially affect any of the observed associations between sweet food propensity and television habits, all of which remained similar in magnitude and significance. Similarly, the association between fat propensity and having a TV in bedroom was not explained by preference for fat in the sensory test. This indicates that taste preference is unlikely to be the mechanism linking television with propensities to consume sugar and to some extent fat. These results are shown in Table 5. The fully adjusted models also indicated no independent associations between high-risk TV behaviors and taste preferences per se (data not shown).

**Table 5:** Relation between television habit and fat/sugar propensity in sub-sample participating in the taste preference tests for sweet and fat

Odds ratio (OR) for television habit, by	Fat propensity				Sugar propensity			
	Model 1^a^		Model 2^b^		Model 1^a^		Model^b^	
	OR^c^	*p* value	OR^c^	*p* value	OR^c^	*p* value	OR^c^	*p* value
Model 1								
EATTV: Regularly eat meals while watching TV	1.20 (N = 1545)	0.0009	1.23 (N = 1255)	0.0006	1.27 (N = 1545)	<0.0001	1.28 (N = 1473)	<0.0001
TV60: At least 60 min/day average weekdays or weekends	1.08 (N = 1571)	0.1342	1.04 (N = 1280)	0.4955	1.20 (N = 1571)	0.0005	1.20 (N = 1469)	0.0006
TVROOM: Has a TV or video/DVD in bedroom	1.12 (N = 1556)	0.0553	1.16 (N = 1266)	0.0302	1.18 (N = 1556)	0.0068	1.19 (N = 1477)	0.0039

^a^Adjusted for age, sex, centre, parental education

^b^Adjusted for age, sex, centre, parental education, fat/sweet preference

^c^Difference for one quartile step

## Discussion

This paper explored potential effects of television exposure among young European children in relation to both weight status and propensity for consuming foods commonly considered to increase risk of obesity. In summary, consumption of high-fat and high-sugar foods were directly and monotonically associated with high-risk television habits, operationalized in terms of time watching TV, eating meals while watching TV, and having TV/video/DVD in the bedroom. The three television indicators were also significantly associated with increased likelihood of being overweight (including obese). None of the associations could be explained by parental educational levels or country in which the survey was conducted. These associations between television habits and childhood weight status are in agreement with much previous literature from Europe and particularly North America, but this is one of the few internationally harmonized studies on the association in young children. There has also been considerable research on the relation between television and unhealthy diet patterns but again there have been few multi-country studies with standardized dietary methodology. In adolescents, there are some international publications revealing associations between media use and daily consumption of sweets, soft drinks, fruits and vegetables [19] and energy dense snacks and beverages [20]. The present work indicates that these dietary associations have their origins earlier in life, and it can be assumed that effects on weight status will accumulate into adolescence and adulthood. These results on TV, overweight and diet quality are thus consistent with current knowledge on this topic. However, the hypothesis that the association between television and preference for fat or sweet foods may be mediated by taste preferences for fatness of sweetness of experimental test-foods [8] could not be confirmed.

Implications of these findings may be considered in two parts. First, we confirmed the excess risk of overweight in young children with more viewing time, having TV in their bedrooms, and who eat meals while watching TV. It is interesting to note that the fully adjusted models predicting overweight with one television indicator simultaneously adjusted for the two other TV indicators. This suggests that all 3 behaviors can be targeted in interventions to prevent obesity. The associations between TV and overweight were robust and not explained by diet, physical activity, or socioeconomic circumstances, and were consistent in most countries included in this study. It cannot be excluded that the international differences in the strength of the association between television habits and overweight may relate in part to between-country differences in regulatory traditions surrounding commercial television aimed at children. However, this issue was beyond the scope of the present study.

In the second part of the paper, we observed that the relation between high-risk television habits and propensity to consume fat- and in particular sugar-containing foods and beverages was quite strong and, in the sub-study of older children, independent of individual taste preferences. This is to our knowledge the first study directly assessing the potential role of taste preferences in mediating the association between TV viewing and diet quality. An apparent dissociation of taste preferences from food choices, which we observed previously [16], resulted in the present observation that the association between TV and diet propensities could not be explained by taste preferences. From this finding it may be inferred that schoolchildren with more TV exposure are consuming high-sugar and high-fat foods *without having a preference for sweet*- *or fat*- *enhanced test foods*, suggesting that passive consumption of these products may be occurring in association with television. It should be noted that our previously mentioned publication [16] observed a direct relation between preference for fat and sugar enriched test foods and overweight in IDEFICS schoolchildren, particularly girls. These previous results, together with those presented here, imply that taste preferences and TV habits may be independent determinants of weight status in children.

Other notable features of this study include its internationally standardized design and methodology as well as a sample size providing statistical power to detect associations of modest magnitude. However, some important methodological issues limit interpretation of the results. For instance, despite standardization of the food frequency questions and some confirmation of their validity and reliability [14, 15], the representativeness of the food frequencies vis-a-vis children’s whole diets may vary due to differences in school meal practices in the different survey settings. Thus in the present analysis it was considered essential to standardize each child’s fat and sugar intakes to the total number of frequencies captured in the questionnaire. Nevertheless, the propensity ratios can only give an approximation of usual fat and sugar intake patterns, and it is possible that complex reporting errors are distorting the role of diet as a mediating factor in the TV-overweight association. In this context, an important source of error derives from the fact that all television viewing and dietary data are by necessity reported by parents or guardians of these young children; in addition to being imprecise, these estimates may be influenced by desire to report healthy habits. For instance, the independence of the TV-overweight association from dietary propensities and physical activity levels is not expected and may in part reflect reporting bias by parents of obese children, as well as residual confounding by other factors. Also when considering limitations, the cross-sectional nature of the data must be emphasized; for this reason we chose to present the “dose-response” of diet propensities in relation to television habits rather than the reverse. These questions will be re-investigated longitudinally when follow-up data become available. Finally, it should be pointed out that the high levels of methodological standardization across survey centers, in which the same instruments were used in different cultural and language contexts, can carry costs in terms of precision. This, together with the fact that survey centers were chosen in each country based on convenience rather than national representativeness, may explain some of the between-center differences in the effect magnitudes illustrated previously.

In conclusion, the present results support the widely held assumption that television plays a role in childhood obesity, while underscoring the complex and multifactorial nature of the problem. These findings may have some practical implications for parents, caregivers and communities attempting health promotion early in life. In particular, the results underscore the necessity of working simultaneously in different behavioral domains, including family meal patterns, types of television use, and availability of sweet and fat foods that are likely to be passively overconsumed by children.
